# Power, control, communities and health inequalities III: participatory spaces—an English case

**DOI:** 10.1093/heapro/daaa059

**Published:** 2020-12-31

**Authors:** Katie Powell, Amy Barnes, Rachel Anderson de Cuevas, Clare Bambra, Emma Halliday, Sue Lewis, Rory McGill, Lois Orton, Ruth Ponsford, Sarah Salway, Anne Townsend, Margaret Whitehead, Jennie Popay

**Affiliations:** 1 School of Health and Related Research (ScHARR), University of Sheffield, Sheffield, UK; 2 Department of Public Health, University of Liverpool, Whelan Building, Liverpool L69 3GB, UK; 3 Institute of Population Health Sciences, Newcastle University, Baddiley-Clark Building, Richardson Road, Newcastle upon Tyne NE2 4AX, UK; 4 Division of Health Research, Lancaster University, Lancaster LA1 4YG, UK; 5 School of Social and Political Science, The University of Edinburgh, Edinburgh, UK; 6 Department of Public Health Environments and Society, London School of Hygiene and Tropical Medicine, 15 − 17 Tavistock Place, London, UK; 7 Department of Sociological Studies, University of Sheffield, Sheffield, UK

**Keywords:** collective control, health equity, determinants of health, participation, communities

## Abstract

This article—third in a series of three—uses theoretical frameworks described in Part 1, and empirical markers reported in Part 2, to present evidence on how power dynamics shifted during the early years of a major English community empowerment initiative. We demonstrate how the capabilities disadvantaged communities require to exercise collective control over decisions/actions impacting on their lives and health (conceptualized as emancipatory power) and the exercise of power over these communities (conceptualized as limiting power) were shaped by the characteristics of participatory spaces created by and/or associated with this initiative. Two main types of participatory spaces were identified: governance and sense-making. Though all forms of emancipatory power emerged in all spaces, some were more evident in particular spaces. In *governance spaces*, the development and enactment of ‘*power to*’ emerged as residents made formal decisions on action, allocated resources and managed accountability. Capabilities for alliance building—*power with*—were more likely to emerge in these spaces, as was residents’ resistance to the exercise of institutional power over them. In contrast, in *sense-making spaces* residents met informally and ‘made sense’ of local issues and their ability to influence these. These processes led to the development of *power within* capabilities and *power to* resist stigmatizing forms of productive power. The findings highlight the importance of designing community initiatives that: nurture diverse participatory spaces; attend to connectivity between spaces; and identify and act on existing power dynamics undermining capabilities for collective control in disadvantaged communities.

## INTRODUCTION

Community *empowerment* comprises processes that develop the capabilities disadvantaged communities need to exercise collective control over decisions and actions that impact their lives and health. We use ‘disadvantaged’ throughout the paper to encompass the multi-dimensional nature of the adverse social and economic circumstances experienced by less privileged communities and neighbourhoods. As a route to political and social transformation for greater equity, community empowerment is enshrined in foundational health promotion texts (WHO, 1997, 1986, WHO EURO, 2013). We use the term health promotion to include practice and policy that in some countries is referred to as public health. In recent decades, these recommendations have been supported by increasing evidence that the extent of collective control communities have is an important determinant of health equity (Wallerstein, 2006; Popay, 2007, 2010; Whitehead *et al.*, 2016). Community empowerment is thus now integral to the Global Sustainable Development Goals and many local, national and international strategies for social and health development (e.g. WHO EURO, 2013; UN Economic and Social Council, 2019; United Nations, 2019). 

In Part 1 of this series of three papers, we argued that, despite this high profile, the processes that support the development of collective control capabilities are not well understood within health promotion (Popay *et al.*, 2020). Drawing on the work of Nussbaum and Sen (Nussbaum and Sen, 1993), we use the term *capabilities* to refer to the *potential* abilities that enable disadvantaged communities to exercise collective control over what they are ‘actually able to do and to be’ and to resist being passively shaped or explicitly controlled by others [(Nussbaum, 2002), p. 129]. We suggest that contemporary community approaches in the health field are increasingly limited to an *inward gaze* on psycho-social capabilities within communities and proximal conditions in neighbourhoods, neglecting an *outward gaze* on political and social transformation for greater equity. We therefore put forward two complementary power frameworks—one focussed on capabilities for collective control, conceptualized as emancipatory forms of power, the other on limiting forms of power—to support health promotion to strengthen this outward gaze in work with disadvantaged communities. In Part 2 of this series, we describe empirical markers of the four dimensions of emancipatory, derived from our evaluation of a large English community empowerment programme, Big Local (BL) (Ponsford *et al.*, accepted for publication). Here, in Part 3 of the series, we use these theoretical frameworks and markers, to present empirical evidence from our evaluation, on the way power dynamics operated in identified spaces for participation during the early years of this 10-year programme. We demonstrate how the emergence of collective control capabilities within BL communities and the relations with forms of limiting power were shaped in different ways by the diverse characteristics of the participatory spaces created by and/or associated with this empowerment initiative.

### Identifying spaces for participation and collective control

The idea of ‘space’ is used widely within diverse literatures on power, policy, international development and collective action (Lefebvre, 1991; Cornwall, 2002; Allen, 2003; Massey, 2005; Gaventa, 2006). Whilst places are typically understood as bounded, singular or with fixed identities, spaces are understood as open and porous, comprised of temporary assemblages of social relations and material products, and formed from interrelations that, crucially, reflect power dynamics over time (Massey, 2005). Massey *et al.* and Massey , in particular, developed space as a unifying concept for analysing the operation and effects of economic, social and political processes (Massey *et al.*, 1976; Massey, 2005). Gaventa similarly draws on ‘space’ to explain how citizens can exert power to create and engage effectively in processes of development at local to global levels (Gaventa, 2006). This 3D understanding of space—as relational, temporal and material—and particularly key elements of these described below—provide a valuable geometry [(cf. (Renedo and Marston, 2015)] for examining the multiple configurations of power (re)produced in community-based initiatives and determining the extent to which communities can develop and exercise collective control over their lives and health.

In the relational dimension, the *impetus* for developing a community-based initiative—its origins and motivations—shape the power dynamics operating within participatory spaces: determining who can enter, with what identity, narrative or interest; what is say-able or do-able; and the relational connections made (Cornwall, 2002; Gaventa, 2006). Cornwall distinguishes between ‘invited’ and ‘claimed’ spaces for participation (Cornwall, 2002). In the former, the impetus for participating is derived by invitation from people in positions of relative power, and existing forms of institutional power (the ‘old rules of the game’) can silence or prevent people from entering. Yet, they can become ‘spaces of possibility’ if previously excluded groups lever access to assert their rights and enhance their influence (Cornwall, 2002). ‘Claimed’ spaces are created by groups who have been marginalized, based on their own terms of participation. *Durability* is a key element of the temporal dimension of participatory spaces. Cornwall differentiates between ‘regularized’ and ‘fleeting’ spaces—characteristics that can affect people’s motivations to engage and the relational practices operating within spaces (Cornwall, 2002). The impact of material dimensions, such as buildings, on participatory spaces in community-based initiatives is well-documented (Butler *et al.*, 2013). A less well-recognized material dimension is the *narratives* of strength and/or resistance (re)constructed in, and shaped by, participatory spaces, which are key elements of empowerment processes (Sommers, 1994; Thomas, 2016; Halliday *et al.*, 2018).

In this paper, we apply these three characteristics—impetus, durability and material—to analyse the power dynamics operating within the participatory spaces that emerged in the BL empowerment initiative, and how these impacted the emergence and exercise of emancipatory power—capabilities for collective control—in communities.

## METHODS

### The study setting: the BL community-based initiative

BL is funded by the English Big Lottery Charity and managed by a not-for-profit organization—Local Trust. This 10+-year initiative involves residents of 150 relatively disadvantaged areas in England receiving £1 million per area to use to improve their neighbourhoods. BL communities did not apply for this funding. Initially the funder produced a long-list of English neighbourhoods that had not received significant lottery funding previously. The final 150 BL areas were selected following discussions between the funder and key stakeholders from local government and the local voluntary and community sector (Local Trust, 2012).

Residents in each neighbourhood decide collectively how to use funds, within a common overall framework set by Local Trust comprising: forming a resident-led BL Partnership; involving the wider community in developing and delivering a local plan; reviewing progress over time and adapting the plan as necessary. BL partnerships are encouraged, but not required, to collaborate with other organizations. The programme is innovative in having the central objective of giving power over the £1 million to residents of BL areas, unlike most previous place-based interventions that give ultimate financial control to local government or other professional institutions. Each BL area had support from a paid BL Rep—people with a range of professional knowledge and experience often in the ‘community’ or not-for-profit sector. Governance over how the money is spent rests with the resident-led Partnership, but, as we describe later, many Partnerships open up the ‘governance space’ to enable the wider ‘community of place’ to contribute to priority setting, decision making and plan delivery.

### Evaluation design

The findings presented here are based on analyses of qualitative data collected during the first phase of our ongoing mixed-method longitudinal evaluation of BL. More details of this study are available at (Communities in Control, 2020). Phase 1 of the evaluation aimed to develop a ‘thick’ description of the first 3 years of the programme. It therefore adopted an interpretative approach utilizing qualitative methods to understand how the programme unfolded through the subjective viewpoints of the residents and other stakeholders involved within their local context. Two waves of fieldwork were conducted between March 2014 and November 2015 in 10 BL areas. These were purposively sampled from the 150 BL areas to provide geographical spread and reflect diversity of local context including: population characteristics, urban/rural, contemporary socio-economic conditions and historical trajectory. The dataset across the ten field-sites included semi-structured face to face interviews with 116 residents and other stakeholders. Interviews explored a priori issues, such as impetus for BL activities, as well as following up incidents identified as significant through other interviews or observations. In addition, participatory activities were conducted (e.g. walkabouts guided by residents) and extensive participant observation of Partnership meetings and other events and informal conversations about people’s experience of BL activities were recorded in structured templates and field notes. Finally, documentary sources (Partnership minutes, plans, website material) were collated and content analysed to provide further insight into the areas and processes through which resident participation was happening. Informed consent was obtained for all fieldwork. Ethical approval was granted by Lancaster University’s Research Ethics Committee (3 February 2014).

Interview transcripts were anonymized, entered into Nvivo 10 and thematically coded using a common broad-brush coding frame, developed iteratively through cross-team discussion so that emerging interpretations were justified (Eisenhardt and Graebner, 2007). This enabled ease of retrieval and cross-referencing during more focussed analysis. For the analysis described here, the dimensions of ‘participatory spaces’ described earlier were used as sensitizing devices to help identify incidents and relationships to code. Analytic memos informed analysis and interpretation (Charmaz, 2006). This involved the use of a systematic template for capturing data relating to how spaces connected to examples of resident-led action. Cross-case analysis was progressed through the sharing of memos and regular face-to-face data analysis workshops with all team members. Analysis continued through a combination of data tabulation and narrative synthesis (Popay *et al.*, 2006), until an overall story had emerged to describe the findings and agreement was reached about a set of general propositions in relation to the cross site data (Yin, 2009).

### Power frameworks

The emancipatory power framework (EPF) and empirical markers of changes associated with each dimension in this framework are shown in Box 1. The EPF comprises three power dimensions reflecting different types of collective control capabilities. The fourth dimension—*Power Over*—refers to situations in which groups may seek to exercise *collective control over* other groups or institutions in the pursuit of change, albeit not always positive for the community as a whole. Relationships between these power dimensions are non-linear—although the exercise of *power to* and *power with* requires some degree of *power within*—and changes in one-dimension feedback into other dimensions.

**Box 1:** Emancipatory power framework and empirical markers in each power dimension

Definition	Power within: capabilities internal to a community supporting collective control/action	Power with: capabilities to build alliances and act with others to achieve common goals	Power to: capabilities to achieve desired ends; includes establishment of structures, procedures and opportunities for collective decisions/actions as well as outcomes of these	Power over: power over other institutions or exercise of power over a group of community members by another group
Empirical markers	*Sharing existing skills/ expertise *Increasing efficacy/confidence in ability to act together *Expressions of shared values, interests and common identity *Developing new collective knowledge, skills and ‘know how’ *Recognition of need for breath/depth of community participation *Arrival at shared vision for area improvements	*Recognition of potential benefits of working with others *Identifying opportunities to develop relationships and/or work with others *Establishing new, or re-shaping previously acrimonious, relationships with others *Inviting local agencies to participate in decision-making/action	*Formation of new inclusive governance structures *Establishing formal practices/frameworks *‘Opening out’ to enable shared decision-making *Improved social, cultural or economic conditions through collective action by residents or influencing decisions of others	*Changes in balance of power to the benefit of community groups *Local politicians/professionals excluded to retain control over decision-making *Lack of transparency in decision making/use of rules/procedures ‘excludes’ others

*Source*: Ponsford *et al.* (Ponsford *et al.*, 2020).

**Box 2:** Limiting power framework: forms of power limit collective control by communities

Compulsory power	Direct and visible exercised by/through, e.g. police, local and national legislation
Institutional power	Less visible, exercised through organizational rules, procedures and norms, e.g. controlling information put into the public sphere, who is involved in decision-making
Structural power	Invisible, systematic biases embedded in social institutions; generating/sustaining social hierarchies of class, gender, ethnicity and resources, opportunities, social status.
Productive power	Invisible, operates through diffuse social discourses and practices to legitimate some forms of knowledge, while marginalizing others. Shapes meanings of different social identities.

*Source*: Popay *et al.* (Popay *et al.*, 2020).

The limiting power framework (LPF), shown in Box 2, identifies four forms of power which can limit the ability of communities to exercise collective control over decisions/actions impacting on their lives. More details on these frameworks and the development of the empirical markers are available in Parts 1 and 2 of this series (Popay *et al.*, 2020; Ponsford *et al.*, 2020).

Codes for the illustrative quotes in the Findings section refer to: fieldwork areas: A1−A10; research method (‘Interview’ or ‘Observation’); participant role (R = resident; BLW = Worker employed by the BL Partnership; LP = Local Politician; LGO= Local Government Officer; PM = Big Local Partnership Member; O = employee of other agencies).

## FINDINGS

Two main types of spaces for participation were identified within BL neighbourhoods: *Governance* and *Sense-making*. In governance spaces we observed the development and implementation of BL policy through formal decision-making, the allocation and use of resources and the management of responsibility and accountability for decisions. Two subtypes of governance space were identifiable. *Partnership spaces* emerged first, as every area established a resident-led Partnership to oversee the planning and delivery of a local area improvement plan. *Project spaces* emerged later, subsidiary to Partnerships, as residents developed specific activities to implement their plans.

In contrast, in sense-making spaces residents met informally and sought to ‘make sense’ of local issues, of BL and their potential to influence these issues collectively. Two sub-types of sense-making spaces were discernible. *Resident spaces* emerged in diverse locations including community hubs, people’s homes, in shops, on the street, or even within breaks during Partnership/project sub-group meetings. *Event spaces* took the form of one-off or repeated events organized by BL Partnerships: fun days, community carnivals, shows and summer galas.

Our findings illuminate two key aspects of power dynamics in these spaces summarized in Box 3. First, they highlight how the impetus for, durability of and narratives (re)constructed within these spaces constituted characteristics that differentially shaped the capabilities—forms of emancipatory power—residents were able to develop. Second, they illuminate how residents collectively exercised these capabilities to resist forms of power being exercised over them and to act to improve their neighbourhoods. BL communities were observed challenging all forms of limiting powers, but for this analysis we focus on resistance to *institutional power*, most evident in governance spaces and to *productive power*, most evident in sense-making spaces.

**Box 3:** Characteristics of, and power dynamics within different types of participatory spaces in BL

Type of space	Sub-type of space	Characteristics of the space			Most evident emancipatory powers developed	Most evident limiting powers resisted
		Relational impetus	Temporal durability	Material narratives		
Governance spaces	Partnership, e.g. meetings associated with BL partnerships	Invited space, becoming more resident claimed over time	Opened up early, with regularized, scheduled participation	Increasingly shaped by the resident-led narratives developing in the resident space	Power to: capabilities to establish opportunities for collective decision-making and to exercise collective control	Institutional power: direct and visible power exercised by/through official channels, e.g. local agencies and professionals trying to control the agenda
	Project, e.g. set up to deliver specific projects or tasks in BL action plans: ‘Tasking Groups’, ‘friends of’ green space groups	Claimed by resident Partnership members and BL workers	Opened up later in BL, participation ebbed and flowed depending on project	A narrative of ‘getting things done’ common	Power with: capabilities to build alliances and act with others to achieve common goals	
Sense- making spaces	Resident, e.g. in community hubs, people’s homes, shops, on the street	Claimed by residents with no set agenda, grounded in residents’ interests	Regular spaces, but spontaneous participation	Where resident-led narratives developed	Power within: capabilities internal to a community supporting collective control/action	Productive power: invisible, systematic biases embedded in social institutions, e.g. stigmatizing narratives about the BL areas perpetuated by media and local agencies
	Event, e.g. one-off/repeat events: fun days, community carnivals, shows, summer galas	Claimed by resident Partnership members and BL workers, motivated by interests in due process	Fleeting spaces with potentially broad participation	Collective understandings of the neighbourhood further developed/shared	Power with (particularly in terms of social connectivity): capabilities to build alliances and act with others to achieve common goals	

### Governance spaces

*Governance spaces* were primarily where the development and enactment of ‘*power to’* emerged, as residents made formal decisions about action to be taken, allocated resources and managed accountability. Capabilities for alliance building—*power with*—were also more likely to emerge in these spaces. Two sub-sets of governance spaces were identified: partnership and project spaces.

#### Partnership spaces and institutional power

The impetus for the formation of BL Partnerships influenced who participated in governance spaces and the practices that dominated them initially. The residents were not required to apply for the funding they received. However, in line with Local Trust’s guidance, every area established a resident-led Partnership to oversee the design and delivery of an action plan. Initially, a BL Rep or a local government employee (i.e. people in established positions of power) invited residents into a ‘new’ space to form a Partnership via public adverts of open meetings or targeted approaches through professionals’ existing links with local groups. This impetus meant residents initially recruited to Partnerships were predominantly affiliated to existing groups, such as a local Tenant/Residents Associations or faith-based groups, and/or had participated in previous neighbourhood initiatives. As a result, the power dynamics between residents, and between residents and local agencies, embedded in early partnership spaces reflected established forms of *institutional power*. Partnership meetings were typically formal and regularized, mirroring the decision-making structures and processes of established fora in the area, particularly those of local government. Some BL Reps and local government workers described how they drew on skills and practices developed in their own organizations (such as voting processes and annual general meetings) to help Partnerships establish governance procedures. Resident Partnership members explained how they came to rely on these practices ‘because you don’t have any alternative, you know there’s… no experience of any alternative’ (A6-I-RPM).

These governance practices could operate as a form of *power over*, excluding some people/groups and/or reducing the community ‘bandwidth’ for developing *power within*. For example, as this quote illustrates, even when invited, some young people experienced Partnership practises and relationships as barriers:

I was reluctant to join the Partnership but the demographic of the Partnership was that I was the youngest one at the time, so people persuaded me to join up… there weren’t pressure, just my fear that I wouldn’t be able to get to many meetings. I feared that if it came down to any votes I might be voted out or that people would not have voted with me so easily because I don’t know that many people (A8-I-RPM)

Similarly, the time commitment of regularized monthly or bi-monthly meetings was reported as off-putting by some residents (e.g. those with caring responsibilities).

#### The development of emancipatory power in partnership spaces

Despite these early dynamics, some partnership spaces did bring together new configurations of residents, creating opportunities for shared learning *(power within)* as this resident described:

I’m still learning so when I’m like hearing sort of different things I’m like, oh, I didn’t really understand that but because it’s just a learning thing for me like, you know, I just have to then realise, OK, you know, I might not understand it today but maybe if someone else explains it to me then tomorrow or something I might get it sort of thing… working in like groups and just listening to like what other people have to say, um, you know, it is such a team building thing because you have to work as a group. (A6-I-RPM)

However, as a Partnership Chair reflected at their annual general meeting the process of developing *power within* ‘*has been difficult at times*’ (A1-O). Additionally, as the quotes below illustrate, Partnership members often reflected on their limited ‘reach’ into the wider community, their representativeness and the extent of professional involvement:

We should have a lot more residents… I think they’re [other residents] quite happy to let the same people do it, but they don’t understand it’s not really for us to be running. And then we worry, conversely, that people think, oh, God, it’s them running everything again… so we worry that that might put people off (A7-I-PM)

At the moment it’s still more the professionals and people we’re employing who potentially take the… I do try in that to ensure that people feel that they’ve got ownership of things. It doesn’t always work (A10-I-RPM)

Over time, involvement in the partnership space could lead to a growing sense of confidence among residents in their collective ability to influence issues locally (*power within*) as this quotation illustrates:

And I thought ‘Well all right I’ll go to a second meeting and see how it is.’ And then all of a sudden it was like ‘Well do you think you might be able to do that?’ and I was like ‘Yeah all right I can do that.’ And slowly I got reeled in and I feel really part of it now. it was that thing of they made me feel valued so I went back because I could see that yes there probably was something I could contribute. And now it’s probably about nine months/ten months down the line and I feel really part of it’ (A10-I-RPM)

In some areas more restrictive criteria for Partnership membership started to be introduced. These procedures were justified in diverse ways, including on grounds of efficiency: ‘*If we add to the Executive in the future, it has got to be somebody that is going to contribute; not just somebody who wants to be on’* (A3-I-RBLW). Whilst such restrictions excluded some, they also strengthened ownership and established a greater sense of legitimacy to act among existing resident Partnership members: enhancing their *power within* and *power to* resist institutional power as this quotation shows:

There’s been a shift from them pushing things, to us taking charge.…. The residents [on the Partnership]…. We had decided that we needed to become a Charity, that changed the atmosphere. So, we decided we were going to become a CIO and we thought right – we’ve now grown up. And we’re gonna take charge (A10-I-RPM)

The formality and governance role of the partnership space also supported the development of *power with* amongst resident members, providing a legitimate forum to engage professionals, whose skills, connections and influence they could use to deliver their plans. In several areas, professionals were ‘invited in’ to formally present to Partnerships including: a builder for A8’s infrastructure project; environmental worker for A9’s green space project; and asset-transfer expert for A1’s community hub project. Over time, some Partnerships established less formal meetings (for example, with play areas for small children) or changed venues to encourage wider participation, reflecting a challenge to the ‘old rules of the game’.

#### Project spaces: ‘getting things done’

The impetus for project spaces was instrumental. They were set up to deliver a specific project or tasks in BL plans; a demonstration of *power to*. Called ‘Tasking Groups’ (A1) and described as showing how *‘everybody is kind of taking different roles’* (A4-I-RPM), these spaces were pervaded with a ‘getting things done’ narrative. They were often assumed by residents to have a shorter lifespan than the partnership space, although some, such as the ‘friends of’ group established in A9 to manage a green space, were expected to continue indefinitely. Participation was voluntary but, in several sites, membership required formal endorsement from Partnerships and terms of reference. In some areas, there were concerns about how inclusive participation was

I don’t think there’s been really enough resident involvement in developing the projects. They’ve been endorsed by the community through publication of the plan… I don’t think we sorted the structures out properly for allowing people to get involved (A10-I-RPM)

Project spaces provided opportunities for a wider group of residents to exercise *power to* act: e.g. to improve local green space (A3, A9), run carnivals (A8) and oversee an asset transfer to establish a community hub (A1). However, at least initially, they also enabled *power within* to develop with residents learning and practicing skills by working together. In A1, for example, the process of working with a paid worker to prepare and submit a business plan to transfer a building to community ownership, allowed residents to learn about asset transfers. Endorsement from, and accountability to, the Partnership also lent legitimacy to residents participating in project spaces: developing a sense that others recognized their capabilities and right to act on their behalf, further enhancing their *power within*. As a resident in A3 explained, her group had been ‘*tasked specifically with… moving the project forward*’. Over time, some Partnerships began to see residents participating in project spaces as ‘experts’, giving them greater responsibility and *power to* influence decisions. In A8, for example, the Partnership Chair noted that residents members of a project space were given authority to approve the business plan for a new community sports facility as ‘*the ones closest to it*’ (A8-O-RPM).

The accountability relationship between partnership and project spaces meant learning was shared via reporting. This enhanced *power within* amongst residents participating in both spaces, as discussions encouraged thinking about how to do things differently and overcome challenges. For example, following low turnout to an action day, residents in A9’s green space project space discussed with Partnership members how to engage different residents more effectively. The skills developed within Project spaces also developed residents’ confidence to exert influence in formal ‘invited spaces’ dominated by institutional power. The quote below illustrates growing confidence amongst two resident Partnership members in their ability to approach a local politician about the failure of local Council to respond to their queries about a new local sports facility the Partnership was developing:

I kept phoning [local government planning department]… “He’s on holiday for 5 or 6 weeks”… so we went to this meeting … and you got by invite so me and [another Partnership member] got invited. So afterwards…this chap were there. He says I’m leader of Council. and [the other Partnership member] says “right I’m having him” (A8-I-RPM)

### Sense-making spaces

Two sub-sets of sense-making spaces emerged: resident and events spaces. These were primarily where *power within*, and to a lesser extent *power with* (especially social connectivity) developed as larger numbers of residents met informally and ‘made sense’ of BL, local issues and their ability to influence these.

#### Resident spaces: developing narratives and connecting spaces

Resident spaces developed in BL hubs established by several Partnerships in libraries or leased shops, for example, or emerged more spontaneously in cafés, streets or resident’s homes. These spaces were ‘claimed’ by a wider group of residents than governance spaces, and characterized by informal and more inclusive practices. Driven by residents’ needs and interests they provided opportunities for discussion of local issues; becoming the primary location for the development of enhanced *power within* the wider community, as the following quotation shows:

People can drop in and out of it [the hub]… and pick up things at their own pace… so the Hub becomes more than just a building, it is something where you know if there’s a problem with, say the council decide to knock down some trees, people sort of say ‘we’ve heard about this and we don’t like it’. (A10-I-RPM)

Within sense-making spaces residents forged deeper interest in, and understanding about, BL. This contributed to an increased sense of residents’ right to participate and facilitated access to Governance spaces, as this resident described:

I got a leaflet through my door saying… “If you want to be part of this thing… come along and you could be elected into the Partnership”. I had no idea what that meant… so I bumped into a friend… “I’ve no idea what I’m doing, I don’t know why I’m doing this” and she’s like, “Well just do it anyway, it’s fine…” so I just went and said my piece. (A6-I-RPM)

The development in sense-making spaces of a narrative around BL as a resident-led initiative served as a vehicle to reshape power dynamics within the partnership space; increasing residents’ confidence to ‘claim’ more collective control over the Partnership’s formal practices. As this note illustrates, we observed residents use the term ‘resident-led’ at Partnership meetings to assert a role in a specific decision being made by the local authority:

There is a discussion on the multi-use games area being developed in the area by the local authority, for which the BL board supported a consultation process with young people… A community worker suggests that BL money can help leverage others’ funds/plans. BLW 02 reinforces – suggests they don’t get into detail on this topic. RPM 11 disagrees: “No, we need to check what’s on their [the local authority’s] plans – this is resident-led, so we’d like to know what’s planned”. (A1-O)

#### Event spaces: resisting stigmatizing productive power

Event spaces provided opportunities for community-wide participation and hence for the development of *power within* amongst larger numbers of residents. Events took the form of public occasions organized and promoted by Partnerships including: village fun days, festivals, shows and galas. These were typically ‘fleeting’ spaces opening up less frequently than resident spaces and with no fixed location or entry requirements. The impetus came from Partnerships wanting to show the wider community ‘*what we are up to*’ (A3-I-RPM) or, as in A4 for example, to encourage people to say what they wanted from BL. These processes enhanced *power within* by giving residents not involved in governance spaces a sense that their voice was heard and supporting greater social connectivity. As a resident Partnership member reflected:

I think it’s sort of benefitted everybody. Certainly, I’ve been going to events there has been a good cross section of people. I wouldn’t say it’s just young or old or anything, it’s pretty much everybody. (A4-I-RPM)

In both types of sense-making spaces, new positive narratives about the area/community were developed or old ones revived and/or sustained, challenging sources of productive power that stigmatized many BL neighbourhoods. The impetus for several ‘fun days’ or ‘galas’ derived from a decision by the Partnership to act to reduce spatial stigma by changing the external perception of their area and several BL plans included actions aimed at changing the reputation of the neighbourhood (Halliday *et al.*, 2018). An annual gala, for example, was described by residents in A8 as an opportunity to make the area ‘what it was’ before the closure of local industry (multiple observations) and festivals organized as part of BL in A4 were described as an opportunity to ‘*rebrand*’ and ‘*change the image*’ of the area (A4-I-RPM).

## DISCUSSION

We identified two main types of participatory space emerging within a major community empowerment initiative underway in 150 areas in England: *governance and sense-making*. The relational, material and temporal characteristics of these spaces shaped practices of inclusion and exclusion, and whether, and if so how, residents’ capabilities for collective control—their *power within*, *power with* and *power to*—developed and were exercised over time.

Governance and sense-making spaces were both ‘spaces of possibility’ [(cf. (Cornwall, 2002)] enabling the development of all three forms of emancipatory power. However, particular forms of power emerged differentially in different types of space. *Governance spaces* were primarily where the development and enactment of *power to* emerged, as residents made formal decisions about taking action, allocated resources and managed accountability. Capabilities for alliance building—*power with*—were also more likely to emerge in these spaces. In contrast, *sense-making spaces* were primarily where *power within*, and to a lesser extent *power with* (especially social connectivity) developed as larger numbers of residents meet informally and ‘made sense’ of BL, local issues and their ability to influence these. Sense-making spaces were therefore important in enabling residents to develop the shared interests and values that provide ‘foundations’ for solidarity and collective action (Ponsford *et al.*, 2020).

The exercise of *institutional power* over residents and their resistance to it was more evident in *governance spaces*. Existing power dynamics between residents, and between residents and local agencies—the ‘old rules of the game’ (Cornwall, 2002)—were reproduced in early partnership spaces*;* with cases of local agencies and professionals trying to control the agenda. However, over time, as residents developed *power within* and in particular a shared understanding of BL as a ‘resident-led’ initiative, they were more able to deployed their own *power over* external agencies in governance spaces. For example, residents rejected or amended the formal regularized procedures for Partnership meetings and memberships initially adopted, thus overriding outside influences. However, while such resistance to *institutional power* might have been experienced by residents within the Partnership as positive/emancipatory, we showed how, at the same time, residents can collectively exercise *power over* other residents in ways that may be experienced as dominating and/or oppressive: for example, by precluding particular people from participating (at all or in particular ways). Further, resident participation in the project space often needed ‘authorization’ by Partnership members; generating concerns, as highlighted in other works, about accountability and representativeness (Cornwall, 2002). Governance spaces then, were not neutral, but characterized by contradictory forms of power that ‘play[ed] across one another’ [(Allen, 2003), pp. 3 − 4; (Gaventa, 2006)].

In contrast, the exercise of *productive power* by external agents (e.g. journalists, professionals) was resisted most obviously by the *power within* that residents developed in *sense-making spaces*. In particular, in some areas, the positive counter-narratives ‘re-constructued’ in these spaces were a powerful resource deployed by residents to challenge prevailing stigmatizing narratives about their neighbourhood (Halliday *et al.*, 2018) as highlighted by others (Chandler and Lalonde, 1998; Williams *et al.*, 2003).

Importantly, ‘fuzzy’ boundaries and interconnections between different types of participatory spaces were important in enabling shifts in power dynamics in these neighbourhoods. The shorter lifespan of project spaces, for example, provided opportunities for a wider group of residents to develop *power within*, in terms of shared skills and confidence and to exercise *power to* act to improve living conditions. In some cases, this led to particular residents being regarded as ‘experts’ and, over time, gaining decision-making responsibility within the Partnership space. Similarly, the *power within* and *power with* that emerged amidst the informality of resident spaces supported residents to engage collectively and in some cases to challenge institutional power in more formal Partnership spaces. In these ways, different types of participatory spaces existed ‘…*in dynamic relationship to one another… constantly opening and closing through struggles for legitimacy and resistance … [with] Power gained in one space through new skills, capacities and experiences… used to enter and affect other spaces*’ [(Gaventa, 2006), p. 27].

### Strengths and limitations

BL is one of the largest community empowerment initiatives to be undertaken in the UK, offering significant opportunities for evaluation. The findings presented here draw on qualitative data collected during the first 3 years of the initiative but our evaluation continues to 2021 enabling us to explore how the power dynamics described develop over time. The findings are also based on data from only 10 sites, which, although a relatively large sample for indepth qualitative research, cannot capture all aspects of diversity across the 150 BL areas. However, we have added a further five sites, purposively sampled to increase the diversity of both local context and planned activities. To our knowledge, this is the first study to integrate analysis of the development of participatory spaces with that of power dynamics in different spaces, providing detailed empirical evidence of increasing collective control capabilities in a substantial community empowerment initiative.

## CONCLUSIONS

This is Part III of a series of three papers reporting on our longitudinal evaluation of a major English community empowerment initiative. We have shown how adding a spatial dimension to the power frameworks developed in Part 1 and empirical markers of power reported in Part II illuminates the situated nature of opportunities emerging in such initiatives for communities to develop the capabilities needed to exercise *collective control* over decisions and actions impacting on their lives and health. Participatory spaces created within these initiatives and the connectivity between spaces, appear significant in supporting the development of different types of emancipatory power in disadvantaged communities and in enabling this power to be used to resist the exercise of power that impacts negatively on the social determinants of health inequalities. Our findings point to the importance of designing community-based initiatives that: nurture a diversity of participatory spaces; attend to connectivity between these spaces; and identify and challenge existing power dynamics that are undermining capabilities for collective control by disadvantaged communities. In particular, initiatives should support the development and sustainability of community-led spaces and support community members to lever greater access to, and influence in, formal governance spaces in which they are marginalized or excluded.
